# CHI3L1 as a Prognostic Biomarker and Therapeutic Target in Glioma

**DOI:** 10.3390/ijms25137094

**Published:** 2024-06-28

**Authors:** Jue Zhou, Dongxu Zhao, Haoyuan Tan, Jin Lan, Yinghui Bao

**Affiliations:** Department of Neurosurgery, Renji Hospital, School of Medicine, Shanghai Jiao Tong University, Shanghai 200127, China

**Keywords:** glioma, CHI3L1, bioinformatics

## Abstract

The role of Chitinase-3-like protein 1 (CHI3L1) in tumor progression has been gradually clarified in different kinds of solid tumors. Hence, we aim to elucidate its prognostic value for glioma. In this study, we analyzed RNA sequencing data combined with corresponding clinical information obtained from The Cancer Genome Atlas (TCGA) and the Chinese Glioma Genome Atlas (CGGA) databases. Differentially expressed genes (DEGs) were acquired based on CHI3L1 expression profiles and were used for Gene Ontology (GO) and Kyoto Encyclopedia of Genes and Genomes (KEGG) analyses. Cox regression, least absolute shrinkage and selection operator (LASSO) regression methods, along with a nomogram, were employed to establish a predictive model. Compared with the corresponding non-tumor tissues, CHI3L1 expression was significantly upregulated in various types of solid tumors, correlating with poor clinical outcomes including glioma. GO analysis identified oxidative stress-related genes (ORGs) that were differentially expressed and modulated by CHI3L1, with 11 genes subsequently identified as potential predictors, using Univariate-Cox regression and LASSO regression. In addition, an index of oxidative stress-related genes (ORGI) was established, demonstrating its prognostic value in conjunction with CHI3L1 expression. The aberrant expression of CHI3L1 was proved in glioma patients through immunohistochemistry (IHC). Meanwhile, the knockdown of CHI3L1 inhibited glioma growth in vitro, and real-time Quantitative PCR (qPCR) confirmed decreased ORG expression upon CHI3L1 knockdown, suggesting the potential prognostic value of CHI3L1 as a therapeutic target for glioma.

## 1. Introduction

Glioma is one of the most lethal tumors and accounts for the majority of central nervous system (CNS) malignancies in adults [1]. A four-grade classification system based on molecular and historical evidence published by the World Health Organization (WHO) is widely acknowledged, categorizing gliomas into WHO grades I–IV and distinguishing between low-degree gliomas (LGGs, WHO grades II–III) and glioblastoma (GBM, WHO grade IV) [2,3]. The overall survival time decreases with an increasing grade; patients with LGGs enjoy a 10-year survival rate of approximately 50% whereas the 5-year survival rate for GBM patients is 5.6% [4,5]. Maximum surgical excision, temozolomide chemotherapy, and radiotherapy are the first-line treatments recommended for most glioma patients [6]. Despite the ongoing development of various treatments such as immunotherapy and tumor-treating fields (TT fields), the outcomes for glioma patients remain grim. Molecular features are continuously used to classify gliomas and are associated with corresponding tumor biology and clinical prognoses. Isocitrate dehydrogenase (IDH) mutation and 1p/19q chromosome co-deletion are positive prognostic factors for patient survival [7,8]. Although molecular profiles have been utilized for classification and treatment, potential heterogeneity exists even within the same category of gliomas in terms of drug resistance and recurrence among other factors. This suggests that the exploration of the molecular characterization of gliomas is still incomplete. Therefore, the identification of molecule characterizations with diagnostic, prognostic predictive, and therapeutic guidance in gliomas remains urgent.

Chitinase-3-like protein 1 (CHI3L1) is one of the members of glycoside hydrolase family 18 with the ability to bind to chitin but without catalytic function [9]. CHI3L1 was originally extracted and identified from osteosarcoma cell line culture medium, and afterwards, it was demonstrated that it can be released by tumor cells, immune cells, stromal-producing cells, etc. [10,11]. Subsequent studies have shown that CHI3L1 expression is significantly elevated in lung and breast carcinomas and its expression is raised after the stimulation of IFN-γ and the Th1 cytokine [12,13,14]. Furthermore, researchers have found that the elevation of local CHI3L1 increases the ability of metastatic tumors to colonize and proliferate, and there is a significant increase in CHI3L1 levels in serum for patients with metastatic breast cancer, indicating the association between CHI3L1 and worse clinical outcomes [15,16]. Moreover, the ability of CHI3L1 to reshape the extracellular matrix (ECM) prevents tumor cells from programmed cell death (PCD) [17]. In addition to neoplasm disorders, an increase in CHI3L1 levels in cerebrospinal fluid (CSF) has been observed in patients with neurodegeneration disorders such as Alzheimer’s disease, suggesting its predictive value in neural disorders [18]. Despite evidence that CHI3L1 has been found to be abnormally expressed in solid tumors and neural disorders and is closely related to patient survival, the role of CHI3L1 in gliomas and the potential mechanism remain undefined. In this study, we aimed to explore the value of CHI3L1 in predicting tumor progress and uncover the potential mechanisms by which CHI3L1 induces poor outcomes in patients with gliomas, providing guidance for the development of targeted treatments in the future.

## 2. Results

### 2.1. High Expression of CHI3L1 in Pan-Cancer and Glioma

**Figure 1:**
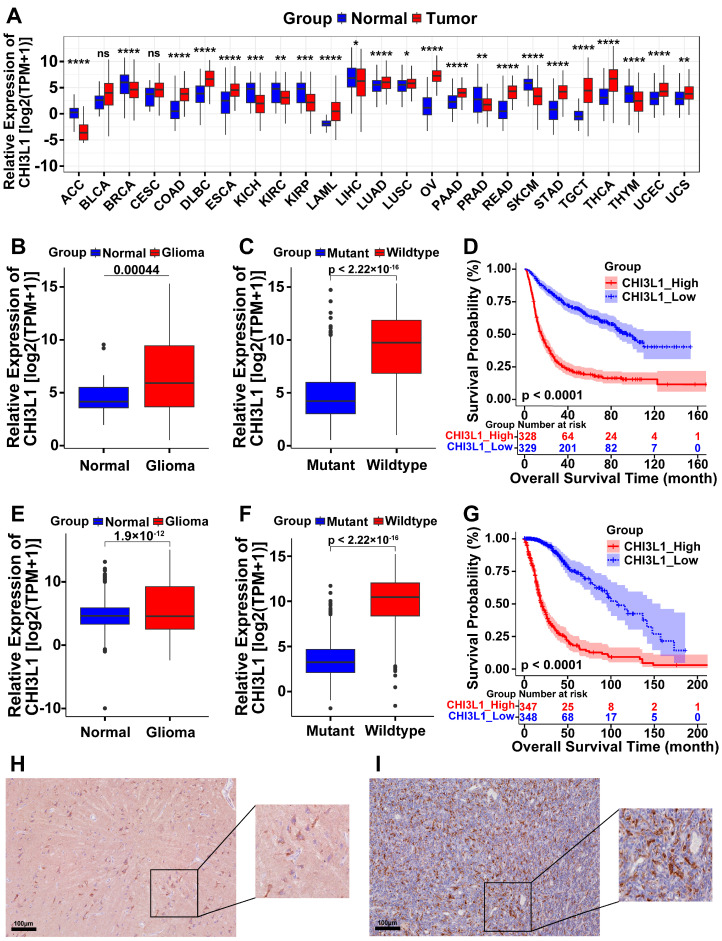
High expression of Chitinase-3-like protein 1 (CHI3L1) in pan-cancer and gliomas. (**A**) Expression of CHI3L1 mRNA in pan-cancer (with significance). (**B**,**E**) Expressions of CHI3L1 in glioma and normal brain tissues in the Chinese Glioma Genome Atlas (CGGA) and The Cancer Genome Atlas (TCGA) cohorts, respectively. (**C**,**F**) Expressions of CHI3L1 varied with different statuses of isocitrate dehydrogenase (IDH) mutation in CGGA and TCGA cohorts, respectively. (**D**,**G**) Overall Survival (OS) of glioma patients with high and low expressions of CHI3L1 in CGGA and TCGA cohorts, respectively. (**H**,**I**) Representative immunohistochemistry (IHC) image of CHI3L1 staining in non-tumor brain tissue and glioblastoma (GBM), respectively (ns, non-significant; *, <0.05; **, <0.01; ***, <0.001; ****, <0.0001).

The relative expression of CHI3L1 mRNA was analyzed in the form of log_2_(TPM + 1) and was observed to be upregulated in various solid tumors compared to their respective normal tissues with statistical significance (Figure 1A). We then examined the transcriptional levels of CHI3L1 in gliomas using data from the Chinese Glioma Genome Atlas (CGGA) and The Cancer Genome Atlas (TCGA) cohorts (Figure 1B,E), revealing significantly higher expression levels of CHI3L1 in glioma samples. Furthermore, elevated CHI3L1 mRNA expression was observed in gliomas with isocitrate dehydrogenase (IDH) status in wildtype samples (Figure 1C,F), indicating a potential association between CHI3L1 expression and poor clinical outcomes. K-M curves were drawn to evaluate the impact of CHI3L1 on patients’ OS (Figure 1D,G), and the patients with lower expression levels of CHI3L1 in gliomas experienced a significantly longer OS than patients with higher expression levels. Immunohistochemistry (IHC) staining performed on non-tumor brain tissue and GBM sections demonstrated a higher concentration of CHI3L1 in GBM (Figure 1H,I).

### 2.2. CHI3L1 Is Involved in Oxidative Stress in Glioma

**Figure 2:**
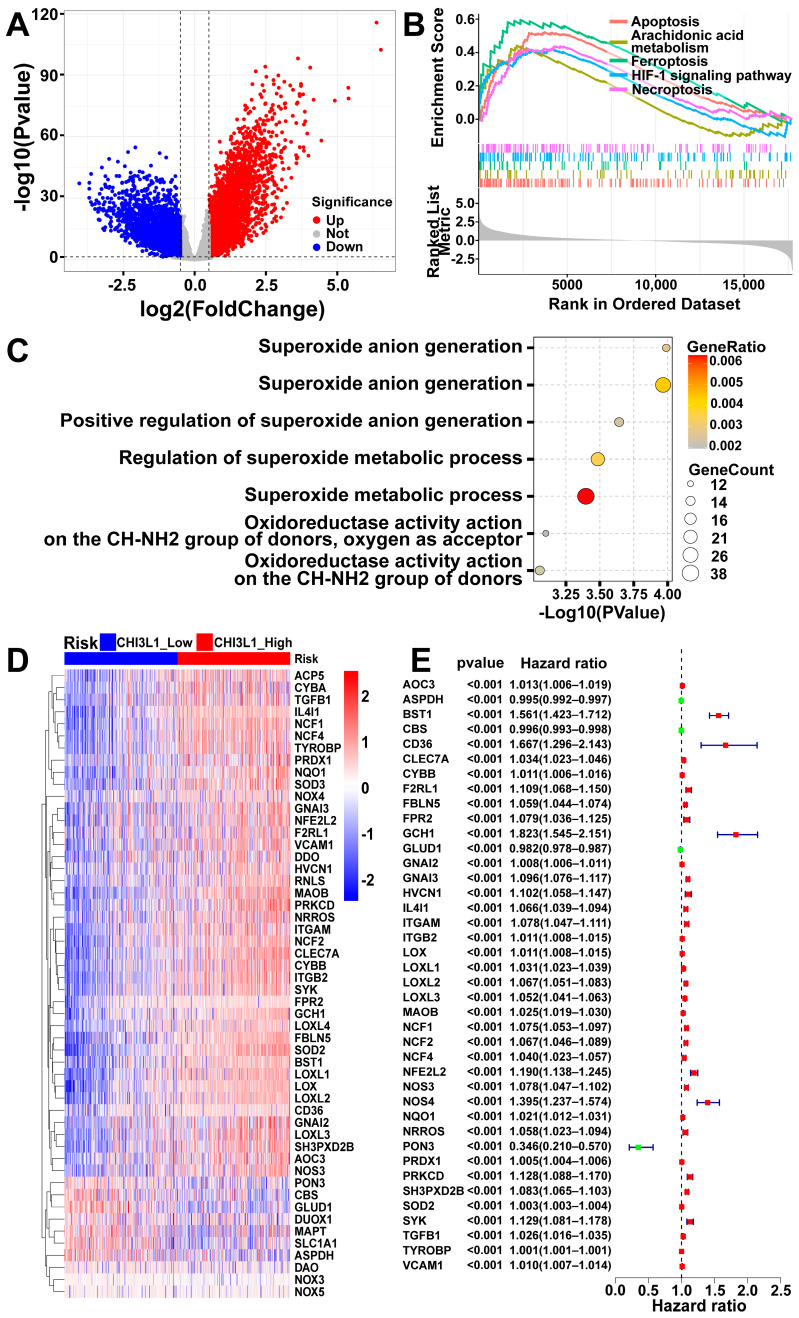
Differentially expressed genes (DEGs) in gliomas based on CHI3L1 expression. (**A**) Volcano plot of DEGs. (**B**) Enrichment of DEGs based on Gene Ontology (GO). (**C**) Gene set enrichment analysis (GSEA) of DEGs based on Kyoto Encyclopedia of Genes and Genomes (KEGG). (**D**) Heatmap of 51genes selected from GO enchainment analysis. (**E**) Univariate Cox regression analysis of 51 genes (with statistical significance).

Gliomas from the CGGA cohort were stratified into CHI3L1 high and low expression groups based on the median mRNA expression levels of CHI3L1 in gliomas. A total of 7059 DEGs were identified by comparing the mRNA expression levels between these two groups (Figure 2A). Enrichment analysis was conducted to elucidate the roles that differentially expressed genes (DEGs) played in gliomas, focusing on Gene Ontology (GO) and Kyoto Encyclopedia of Genes and Genomes (KEGG) pathways (Figure 2B,C). The identified biological processes (BPs), molecular functions (MFs), and pathways were found to be closely related to oxidative stress and programmed cell death. Specifically, 52 genes related to the regulation of superoxide anion generation, superoxide metabolic process, and oxidoreductase activity were selected and visualized in a heatmap (Figure 2D) to illustrate the expression patterns of these genes across different samples. We hypothesized that the DEGs might have implications for patient survival. To investigate this, Univariate Cox regression analysis was conducted to assess the association between the expression of these genes and patient survival. Out of the fifty-two selected genes, forty-one were found to be significantly associated with patient survival, including four protective factors and thirty-seven risk factors (Figure 2E).

### 2.3. Identification of an 11-Gene Risk Index Associated with Oxidative Stress

**Figure 3:**
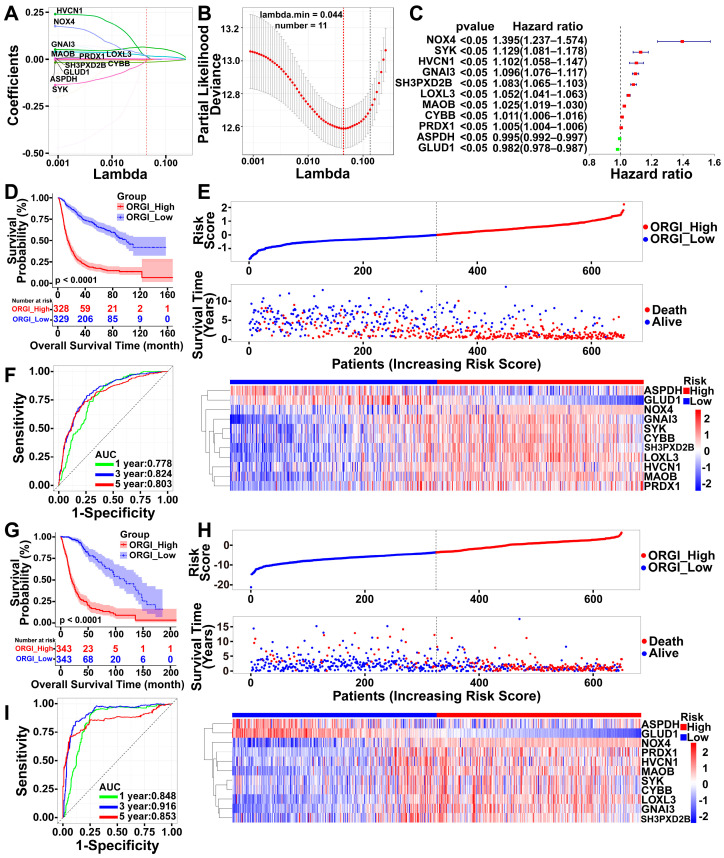
Survival analysis of index of oxidative stress-related gene (ORGI) risk. (**A**,**B**) The minimum selection criteria of the LASSO regression. (**C**) Forest plot of 11 genes selected through the least absolute shrinkage and selection operator (LASSO) regression methods. (**D**,**G**) OS of glioma patients with high and low risks of ORGI. (**E**,**H**) The distribution plots of ORGI risk score, survival status of glioma patients, and heatmap of ORGI in CGGA and TCGA cohorts, respectively. (**F**,**I**) ROC curves of ORGI risk for 1-, 3-, and 5-year OS prediction.

Although the initial set of 41 genes showed promise as individual predictors for prognostic assessment, a LASSO regression analysis was applied to further refine the selection of genes and enhance the model’s accuracy (Figure 3A,B). A total of 11 genes (NOX4, SYK, HVCN1, GNAI3, SH3PXD2B, LOXL3, MAOB, CYBB, PRDX1, ASPDH, GLUD1) were screened out for inclusion in the prognostic model. Among these, two were considered protective factors while nine were hazard factors (Figure 3C).

The oxidative stress-related gene (ORGI) risk score was calculated for each patient in the CGGA cohort, serving as the training set. Patients were clarified into two distinct groups in view of the median ORGI risk score, and the K-M curve proved the predictive efficacy of the risk score in estimating patient survival time (Figure 3D). Survival status and ORGI expression were plotted with increasing risk scores or ORGI subgroups (Figure 3E).

Additionally, assessments on the specificity and sensitivity of the risk scores to predict pathological features with the ROC curves were determined by calculating the areas under the curve (AUCs) of the ORGI risk scores (Figure 3F). Affirmatively, the risk score was capable of predicting the survival status effectively (5-year AUC = 0.803, 3-year AUC = 0.824, 1-year AUC = 0.778). Moreover, the experiments above, from the K-M curve to the ROC assessment, were repeated in the TCGA cohort, yielding similar conclusions (Figure 3G–I).

### 2.4. Prognostic Value of CHI3L1 and ORGI for Glioma Patients

**Figure 4:**
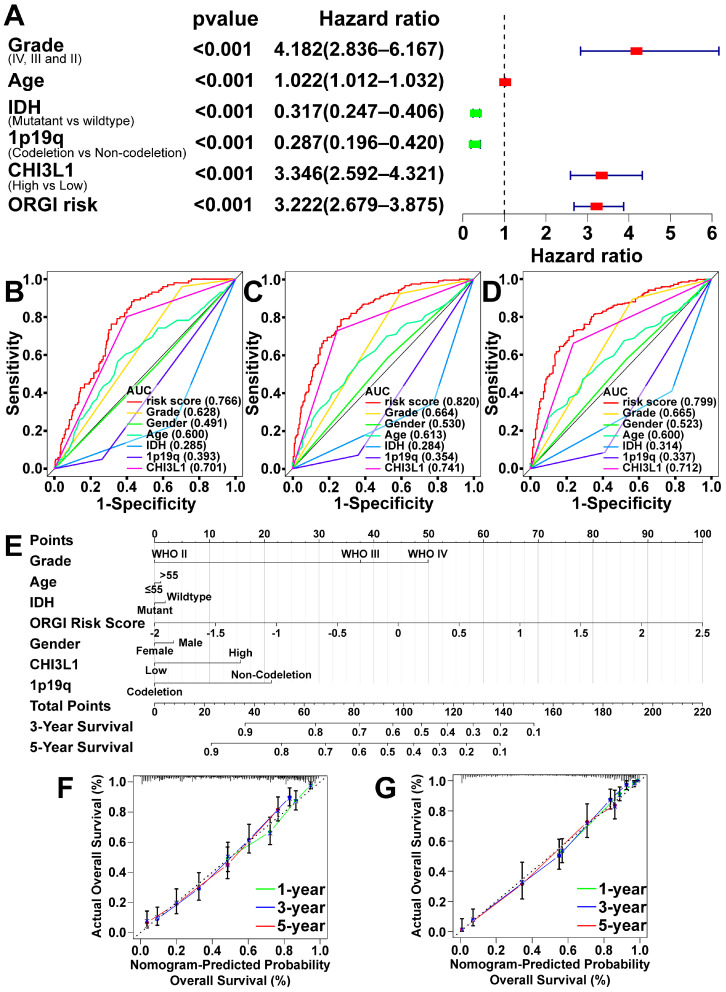
Prognostic model construction. (**A**) Multivariate Cox regression analysis for ORGI risk score, CHI3L1, and clinical status (grade, age, IDH mutation status, co-deletion status of 1p/19q chromosomes) (CGGA cohort). (**B**–**D**) Receiver operating characteristic (ROC) curves of ORGI risk and clinical factors for 1-, 3-, and 5-year OS prediction (CGGA cohort). (**E**) Prognostic model of ORGI risk score, expression of CHI3L1, and clinical factors in nomogram (CGGA cohort). (**F**,**G**) Calibration curves showing the concordance between predicted and observed 1-, 3-, and 5-year OS in CGGA and TCGA cohorts.

To comprehensively assess the impact of CHI3L1 and ORGI on patients’ clinical outcomes, a multivariable Cox regression analysis was adapted, considering patients’ clinical statuses (Figure 4A). The analysis revealed that tumor grade, age, CHI3L1, and ORGI were identified as risk factors with HR 4.18, 1.022, 3.346, and 3.222, respectively, all with *p*-values of less than 0.001. Conversely, the mutation status of IDH and the co-deletion status of 1p/19q chromosomes were protective factors with HR 0.317 and 0.287, both with *p*-values of less than 0.001. The ROCs were also demonstrated for specificity and sensitivity measurement (Figure 4B–D). Subsequently, a nomogram was constructed based on these factors, providing quantitative points for predicting 3- and 5-year OS (Figure 4E). The C-indices of the nomogram in the CGGA cohort were 0.77 ± 0.013, and they were 0.86 ± 0.010 in the TCGA cohort (Figure 4F,G).

### 2.5. Knockdown of CHI3L1 Inhibited Proliferation of Glioblastoma Cells In Vitro

**Figure 5:**
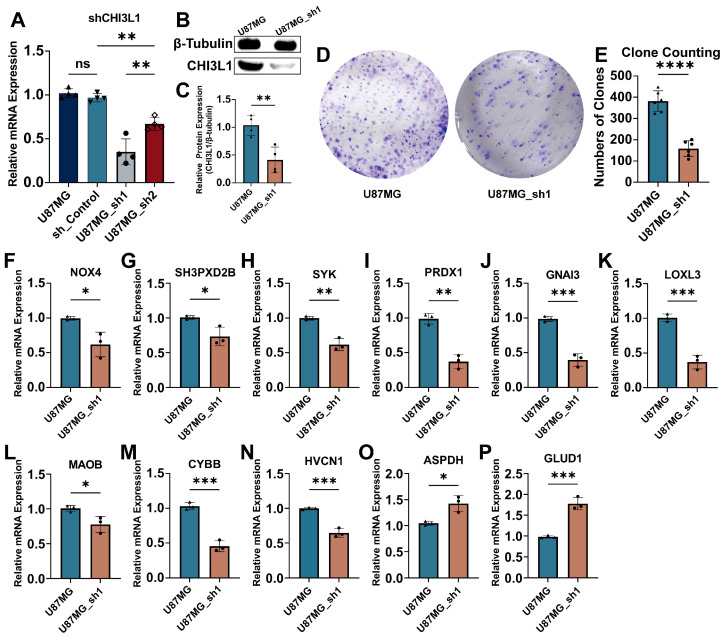
Function verification of CHI3L1 in vitro. (**A**) Verification of silencing efficacy of CHI3L1 shRNA using qPCR. (**B**,**C**) Protein quantity results of CHI3L1 knockdown in U87MG. (**D**,**E**) Colony formation assay for evaluation of proliferation between U87MG and U87MG_sh1. (**F**–**P**) qPCR assay for identification of ORGI mRNA regulation after CHI3L1 knockdown (NOX4, SH3PXD2B, SYK, PRDX1, GNAI3, LOXL3, MAOB, CYBB, HVCN1, ASPHD, GLUD1) (ns, non-significant; *, <0.05; **, <0.01; ***, <0.001; ****, <0.0001).

To validate the function of CHI3L1 in glioma progression, we silenced CHI3L1 in the U87MG cell line (U87MG_sh1). The knockdown efficacy was detected through qPCR assay and Western blotting (Figure 5A–C). A colony formation assay was then carried out for identifying the proliferation capacities of U87MG cells and U87MG_sh1 (Figure 5D), which demonstrated a significant inhibition of proliferation in U87MG_sh1 cells. Furthermore, the expression levels of 11 genes of ORGI were tested via qPCR (Figure 5F–P), showing that NOX4, SH3PXD2B, SYK, PRDX1, GNAI3, LOXL3, MAOB, CYBB, and HVCN1 were downregulated upon CHI3L1 knockdown while ASPDH and GLUD1 were the opposite. The functional verification was performed in U251MG, resulting in the same conclusion (Supplementary Figure S1).

## 3. Discussion

The complex genetic landscape of gliomas poses significant challenges for our comprehensive understanding, as well as for the development of treatments. Gene expression data from the microarray and bulk sequencing of glioma specimens obtained from patients were collected from the TCGA and CGGA databases separately, with 1397 independent datasets in total. We observed that the expression of CHI3L1 was consistently upregulated in various solid tumors, contributing to poor clinical outcomes, indicating its distinctive role in tumor progression, particularly in gliomas. Previous studies have linked CHI3L1 to the activation of inflammation, with researchers also noting its regulation of cytokines and proinflammatory mediators in tumors such as Interleukin (IL)-8(IL-8) and CXCL2 [19,20]. Further investigations revealed that CHI3L1 plays a role in reshaping the tumor microenvironment by promoting angiogenesis and inducing the transformation of tumor-associated macrophages (TAMs) and neutrophils (TANs) [21]. While previous studies mainly focused on the relationship between CHI3L1 and immunity, in this study, we explored the mechanisms underlying the protein interaction network of CHI3L1. Our work identified the prognostic value of CHI3L1 in gliomas, suggesting it is an adverse factor for patients. Further studies have shown that CHI3L1 can alter the oxidative stress in the tumor microenvironment, significantly diminishing the OS of patients. By shrinking the hazard factor scale through regression analysis, we identified a index of 11 oxidative stress-related genes (ORGI), including NOX4, SYK, HVCN1, GNAI3, SH3PXD2B, LOXL3, MAOB, CYBB, PRDX1, ASPDH, and GLUD1.These genes are adjusted by CHI3L1, which can influence patients’ survival expectations. NOX4 and CYBB (NOX2) are members of the NADPH oxidase (NOX) family, being responsible for reactive oxygen species (ROS) production and widely considered to play a major role in immune defense [22]. Previous studies have shown that NOX4 has the potential to promote the progression of non-small cell lung cancer (NSCLC), colorectal cancer, and pancreatic cancer while CYBB has been approved to enhance the metastasis of melanoma in mice [23,24,25,26]. In addition, tyrosine kinase substrate with four SH3 domains transcribed from the SH3PXD2B (TKS4) gene selectively supports distinct NOXs, including NOX1 and NOX3, which contributes to the assembly of NOXs and NOX-associated ROS formation, although the equivalent effect was not observed in the interaction with NOX4 and CYBB [27]. The PRDX1 gene encodes a member of the peroxiredoxin family of antioxidant enzymes and can inhibit the mitochondrial apoptosis in liver cancer, accelerating its progression [28]. Additionally, it has been observed to be highly expressed in NSCLC and triple-negative breast cancer (TNBC) [29,30]. Computational biology and in vivo verification have elaborated on the potential of MAOB to activate the CXCR4-Src/JNK signaling pathway, eventually aiding in the growth and progression of prostate cancer [31]. Another protein acts as an amine oxidase, and additionally, the lysyl oxidase is encoded by the LOLX3 gene. The depletion of LOLX3 can rescue the sensitivity of liver cancer towards chemotherapy via the activation of ferroptosis [32]. GNAI3 was identified as a biomarker indicating the relative risk of susceptibility to gastric cancer [33]. 

Redox reactions always occur in cells and are closely related to homeostasis, inflammation, and immune maintenance in the body [34,35]. Oxidative stress is mostly caused by ROS and is prevalent in senescent cells, inflammatory cells, immune cells, and tumor cells [36,37]. Although ROS are toxic and are often thought to impair cellular homeostasis and may ultimately lead to cell death, they are also observed in tumor cells and are pro-carcinogenic to this extent. Tumor cells may benefit from the proliferative effects of ROS while avoiding ROS toxicity by enhancing their antioxidant systems [38,39]. 

In our study, we elucidated the prognostic predictive value of CHI3L1 in gliomas and identified the relationship between CHI3L1 and increased levels of local oxidative stress in gliomas through DEG and pathway enrichment analysis. We simulated and anticipated the adverse effects of ORGI on glioma patients using prognostic prediction models. To date, most glioma studies have focused on single-gene target finding and functional exploration, which do not fully reflect the genetic complexity and tumor biology of gliomas. In our study, we explored the predictive value of CHI3L1 in gliomas and constructed a prediction model based on CHI3L1, which is aberrantly expressed in a variety of solid tumors and neurodegenerative lesions. Subsequent in vivo and cellular experiments confirmed the expression levels of CHI3L1 in gliomas and their correlation with ORGI. Therefore, the prognostic signature based on CHI3L1 and ORGI might hold a potential position in the development of treatment for gliomas.

## 4. Materials and Methods

### 4.1. Data Collection

RNA sequencing data and corresponding clinical records of glioma patients (*n* = 702) were obtained from The Cancer Genome Atlas (TCGA) database (Cohort: TCGA lower-grade glioma and glioblastoma, https://xenabrowser.net/datapages/ or https://portal.gdc.cancer.gov/, accessed on 28 February 2024) and the Chinese Glioma Genome Atlas (CGGA) database (*n* = 693) (DataSet ID: mRNAseq_693, http://www.cgga.org.cn/, accessed on 28 February 2024), with data from normal brain tissues drawn from CGGA (*n* = 20) (DataSet ID: mRNA sequencing (non-glioma as control), accessed on 28 February 2024). Pan-cancer RNA sequencing data were acquired from TCGA (*n* = 10,496, Supplementary Table S1) and RNA sequencing data of normal tissues from the Genotype-Tissue Expression (GTEx) database (*n* = 7858) (GTEx Analysis V8, https://www.gtexportal.org/home/downloads/adult-gtex/bulk_tissue_expression, accessed on 28 February 2024). All sequencing data obtained were either in the form of transcripts per million (TPM) or processed into TPM for further analysis.

### 4.2. Deferential Expression and Survival Analysis

The expression of CHI3L1 was identified in the pan-cancer cohort, TCGA, and CGGA using R (https://www.r-project.org/). Boxplots and Kaplan–Meier (K-M) curves were generated using the ‘survminer’ package. Differential expression analysis was conducted using the ‘limma’ package based on the expression of CHI3L1 in the CGGA cohort, with significance criteria at a *p*-value < 0.05 and |log2 fold change| > 0.5.

### 4.3. Functions and Pathway Enrichment Analysis

Gene Ontology (GO) and Kyoto Encyclopedia of Genes and Genomes (KEGG) analyses were performed on the differentially expressed genes (DEGs) identified from the CGGA cohort using the ‘clusterProfiler’ package with the criteria of *p*-value < 0.05 and |log2 fold change| > 1, aiming to identify the biological processes, molecular functions, and pathways regulated by CHI3L1 through gene set enrichment analysis (GSEA).

### 4.4. Index of Oxidative Stress-Related Genes (ORGI) and Prognostic Value

Following a detailed review of the functions and pathways enriched after GO and GSEA analyses, 52 genes were selected via GO functional enrichment analysis, pertaining to five bioprocesses and two molecular functions. Univariate Cox regression and least absolute shrinkage and selection operator (LASSO) regression were conducted to determine gene dimensions, resulting in an 11-gene index of ORG. The index was further validated and presented as a forest plot with hazard ratios (HRs) and *p*-values using the ‘survival’ package.

### 4.5. Establishment and Validation of ORGI Prognostic Model

An 11-gene ORGI was identified through Uni-cox and LASSO regressions using CGGA as the training cohort with their regression coefficients (coef). The ORGI risk score was calculated using the following formula:

(1) $$ORGI\;Risk\;Score=\sum_{i=1}^{n}{Coef}_{i}\times x_{i}$$

Here, the ${Coef}_{i}$ and $x_{i}$ refer to the expressions of the indicated genes and their corresponding coef values, respectively. Patients were then stratified into higher- and lower-risk groups based on their ORGI risk scores, with the median value serving as the cut-off. The K-M curve was used to compare the overall survival (OS) between the subgroups. Additionally, a heatmap and survival conditions were presented to illustrate the expression and prognostic value of ORGI with increasing risk scores. Receiver operating characteristic (ROC) curves were plotted using the ‘timeROC’ package to assess the efficacy of ORGI risk scores in predicting patient outcomes. All experiments were initially conducted in the training cohort of CGGA and subsequently validated in TCGA cohort.

Multivariate Cox regression analysis and nomogram were exerted to construct a model incorporating multiple clinical risk factors and ORGI risk scores to comprehensively predict OS. Concordance indices (C-indices) and calibration curves were constructed to evaluate the performance of the nomogram in both the CGGA and TCGA cohorts.

### 4.6. Clinical Case Validation

Specimens of three GBM and three non-tumor tissues were collected from patients who underwent resection for medical needs at Shanghai Renji Hospital, along with their respective clinical data. Glioma specimens were histologically diagnosed as GBM. The Ethics Committee of Shanghai Renji Hospital approved this study, and written informed consent was obtained from all participants. The collected tissues were fixed by immersion in 4% paraformaldehyde for 48 h and in 70% ethanol for approximately 24 h. The dehydrated tissues were then embedded in paraffin and sectioned into four-micrometer slices for immunohistochemical staining. The following steps were used for slice dewaxing and hydration: (I) three washes with xylene for 10 min each and (II) immersion in 100%, 95%, and 75% ethanol for 2 min each. Afterwards, the slices were boiled in 0.01 M citric acid solution for 4 min and allowed to cool to room temperature for three cycles. A 3% hydrogen peroxide solution was used to inactivate endogenous catalase, followed by incubation in 1% Triton-X 100 for penetration and 5% Bovine albumin (BSA) for blocking. The CHI3L1 rabbit anti-human antibody (12036-1-AP) was purchased from Proteintech (Wuhan, China). Slices processed were incubated with primary antibody at 4 °C overnight, followed by incubation with a secondary antibody labeled with horseradish peroxidase (HRP) at room temperature for 30 min. A brown precipitate resulting from the reaction of diaminobenzidine (DAB) with HRP in the sections was used to evaluate the target gene while the cell nuclei were counterstained blue–purple with hematoxylin for 30 s. Finally, the dehydration procedure was repeated and the slices were sealed with a neutral resin.

### 4.7. Cell Culture

The human glioma cell line U87MG was purchased from the Chinese Academy of Sciences Cell Bank (CASCB, Shanghai, China) with certificate of short tandem repeat (STR) appraisal. The cells were cultured in Dulbecco’s modified Eagle’s medium (DMEM) (Gibco, Rockville, MD, USA) with 10% fetal bovine serum (Gibco), 100 mg/mL streptomycin, and 100 IU/mL penicillin and cultivated in 5% CO_2_ at 37 °C.

### 4.8. Real-Time Quantitative PCR (qPCR)

Total RNA was extracted from cells using TRIzol (R0016) (Beyotime, Shanghai, China), chloroform, isopropanol, 75% methanol, and DEPC water. The HiScript II 1st Strand cDNA Synthesis Kit (Vazyme, Nanjing, China) was utilized for cDNA synthesis. Finally, qPCR assays were conducted using AceQ qPCR SYBR Green Master Mix (Vazyme, Nanjing, China) on S1000 Thermal Cycler (Bio-Rad Laboratories, Hercules, CA, USA) following the cycling program: 95 °C for 5 min (once), then 95 °C for 10 s plus 60 °C for 30 s (30 cycles). The relative expression of target genes was calculated and shown in the form of 2^−ΔΔCt^. The primer sequences used are listed in Table 1.

### 4.9. CHI3L1 Knockdown In Vitro

Human CHI3L1 shRNA plasmids were purchased from GenePharma (Shanghai, China), comprising both control and target shRNA sequences as follows: control shRNA—5′-TTC TCC GAA CGT GTC ACG T-3′, CHI3L1 shRNA1:5′-CAAGGAAATGAAGGCCGAATT-3′, and CHI3L1 shRNA2:5′-CCTGACAGATTCAGCAACACT-3′. The U87MG cell line was selected for transfection with shRNA plasmids using Lipofectamine 3000 Transfection Reagent (L3000015) (Invitrogen, Carlsbad, CA, USA). qPCR and Western blotting were performed to measure the knockdown efficacy of plasmids.

### 4.10. Western Blotting

RIPA lysis buffer (Thermo Fisher, Rockford, IL, USA) combined with a Protease Inhibitor Cocktail (Sigma-Aldrich, Darmstadt, Germany) was used for tissue digestion and total protein extraction. Protein concentration was assessed through the BCA method to ensure uniform protein content among the samples. Metal bath at 95 °C for 10 min followed dilution of protein solution with 5×sodium dodecyl sulfate (SDS)–PAGE protein loading buffer. Proteins were separated by 12% SDS–polyacrylamide gel electrophoresis and transferred to a polyvinylidene difluoride (PVDF) membrane (Bio-Rad Laboratories, Hercules, CA, USA). The membrane was incubated with primary antibody solution at 4 °C overnight after 1.5 h block with 5% skim milk at room temperature. Tris-buffered saline with 0.1% Tween-20 (TBST) was used for membrane washing thrice for 10 min at room temperature before incubation with secondary antibodies for 3 h at room temperature. Protein visualization was achieved using BeyoECL Moon luminous liquid (Beyotime, Shanghai, China). Protein band intensities were analyzed using ImageJ software (1.54i, 03 March 2024, Bethesda, MD, USA).

### 4.11. Colony Formation Assay

Cells at a density of 80–100% in flasks were detached with trypsin into single cells and washed with phosphate-buffered saline (PBS). The suspended cells were diluted to the appropriate concentration and plated at 500 cells per well. Plates were placed in an incubator with 5% CO_2_ at 37 °C. The medium was changed every two days until the seventh day after plating. Cells were fixed with 4% paraformaldehyde solution (PFA) for 15 min, followed by staining with 0.5% crystal violet solution for colony visualization. Colony counting was performed using the ImageJ software, and the data were analyzed using GraphPad Prism 9 (Boston, MA, USA).

## 5. Conclusions

In summary, this study investigated the relationship between CHI3L1 and glioma progression and constructed a prediction model based on CHI3L1 and oxidative stress. These findings provide new insights into the treatment and prognosis of gliomas and contribute to the development of novel therapeutic targets.

## Figures and Tables

**Table 1:** Primer list.

Gene Symbol	Sequences
CHI3L1	Forward sequence (5′→3′) GTGAAGGCGTCTCAAACAGG Reverse sequence (5′→3′) GAAGCGGTCAAGGGCATCT
GAPDH	Forward sequence (5′→3′) GGAGCGAGATCCCTCCAAAAT Reverse sequence (5′→3′) GGCTGTTGTCATACTTCTCATGG
NOX4	Forward sequence (5′→3′) CAGATGTTGGGGCTAGGATTG Reverse sequence (5′→3′) GAGTGTTCGGCACATGGGTA
SYK	Forward sequence (5′→3′) CATGGAAAAATCTCTCGGGAAGA Reverse sequence (5′→3′) GTCGATGCGATAGTGCAGCA
HVCN1	Forward sequence (5′→3′) CACCCACACCAGTCTCAGG Reverse sequence (5′→3′) TGTCGGGCTGGATGATCTTCA
GNAI3	Forward sequence (5′→3′) ATCGACCGCAACTTACGGG Reverse sequence (5′→3′) AGTCAATCTTTAGCCGTCCCA
SH3PXD2B	Forward sequence (5′→3′) TGGAGGTGAAGGTGCTAGAC Reverse sequence (5′→3′) CTGTAGCGCCGGTAAATGG
LOXL3	Forward sequence (5′→3′) GATACAGCGAGCTGGTGAATG Reverse sequence (5′→3′) CATCCTCATCGTGCGTACAGT
MAOB	Forward sequence (5′→3′) GGCGGCATCTCAGGTATGG Reverse sequence (5′→3′) GGTCTCCAATCCTAGCTCCTTG
CYBB	Forward sequence (5′→3′) ACCGGGTTTATGATATTCCACCT Reverse sequence (5′→3′) GATTTCGACAGACTGGCAAGA
PRDX1	Forward sequence (5′→3′) CCACGGAGATCATTGCTTTCA Reverse sequence (5′→3′) AGGTGTATTGACCCATGCTAGAT
ASPDH	Forward sequence (5′→3′) GCACTGTGCTCTACGAAGG Reverse sequence (5′→3′) ATCACCCCATCGAAGCCCA
GLUD1	Forward sequence (5′→3′) ACAGTCCAAAGAATGCAGTCAC Reverse sequence (5′→3′) CAGGTGAGTAGGGGCCATTG

## Data Availability

Publicly available datasets were analyzed in this study. Publicly available data were obtained from the official TCGA websites (https://portal.gdc.cancer.gov/ or https://xenabrowser.net/datapages/, accessed on 28 February 2024), GSEA website (https://www.gsea-msigdb.org/gsea/index.jsp, accessed on 28 February 2024), GO website (http://geneontology.org/, accessed on 28 February 2024), CGGA website (http://www.cgga.org.cn, accessed on 28 February 2024), and GTEx website (https://www.gtexportal.org/home/, accessed on 28 February 2024).

## Supplementary Materials

The following supporting information can be downloaded at: https://www.mdpi.com/article/10.3390/ijms25137094/s1.
