# Supplementary material for: CHI3L1 as a Prognostic Biomarker and Therapeutic Target in Glioma
Source: Int J Mol Sci. 2024 Jun 28;25(13):7094. doi: 10.3390/ijms25137094 (PMC11240893; doi:10.3390/ijms25137094)
Supplement: Supplementary file 1 [file ijms-25-07094-s001.zip › Supplementary Materials/Supplementary Figure S1/Supplementary Figure S1.pdf]

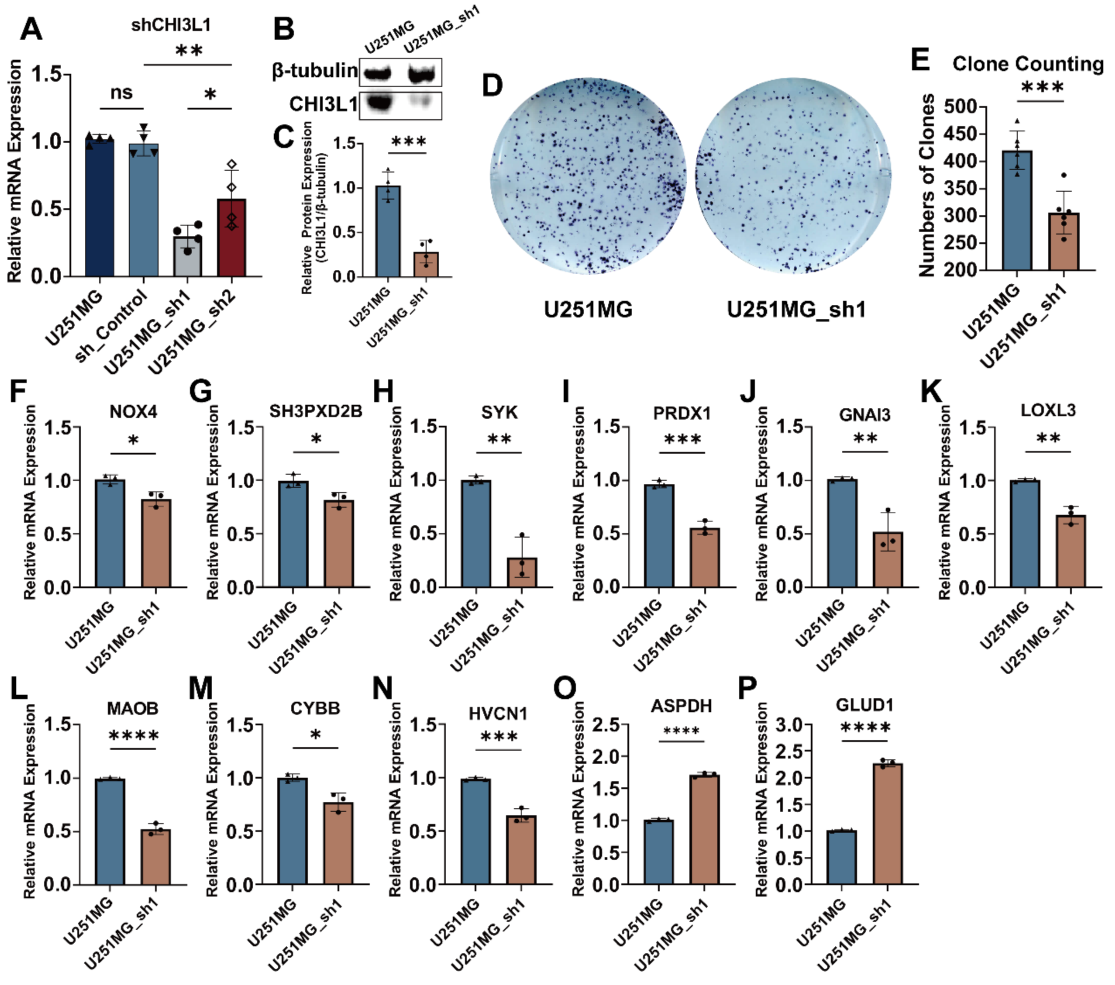

Figure 5. Supplementary Function verification of CHI3L1 in vitro. (**A**) Verification of silencing efficacy of CHI3L1 shRNA using qPCR; (**B**,**C**) Protein quantity results of CHI3L1 knockdown in U251MG; (**D**,**E**) Colony formation assay for evaluation of proliferation between U251MG and U251MG_sh1; (**F**–**P**) qPCR assay for identification of ORGI mRNA regulation after CHI3L1 knockdown (NOX4, SH3PXD2B, SYK, PRDX1, GNAI3, LOXL3, MAOB, CYBB, HVCN1, ASPHD, GLUD1). (ns, non-significant; *, < 0.05; **, < 0.01; ***, < 0.001; ****, < 0.0001).
