# Supplementary material for: CHI3L1 as a Prognostic Biomarker and Therapeutic Target in Glioma
Source: Int J Mol Sci. 2024 Jun 28;25(13):7094. doi: 10.3390/ijms25137094 (PMC11240893; doi:10.3390/ijms25137094)
Supplement: Supplementary file 1 [file ijms-25-07094-s001.zip › Supplementary Materials/Supplementary Table S1.pdf]

	**Pancer Cohort Name**
1	ACC
2	BLCA
3	BRCA
4	CESC
5	CHOL
6	COAD
7	DLBC
8	ESCA
9	HNSC
10	KICH
11	KIRC
12	KIRP
13	LAML
14	LIHC
15	LUAD
16	LUSC
17	MESO
18	OV
19	PAAD
20	PCPG
21	PRAD
22	READ
23	SARC
24	SKCM
25	STAD
26	TGCT
27	THCA
28	THYM
29	UCEC
30	UCS
31	UVM
