# Supplementary figures and images for: CHI3L1 as a Prognostic Biomarker and Therapeutic Target in Glioma
Source: Int J Mol Sci. 2024 Jun 28;25(13):7094. doi: 10.3390/ijms25137094 (PMC11240893; doi:10.3390/ijms25137094)

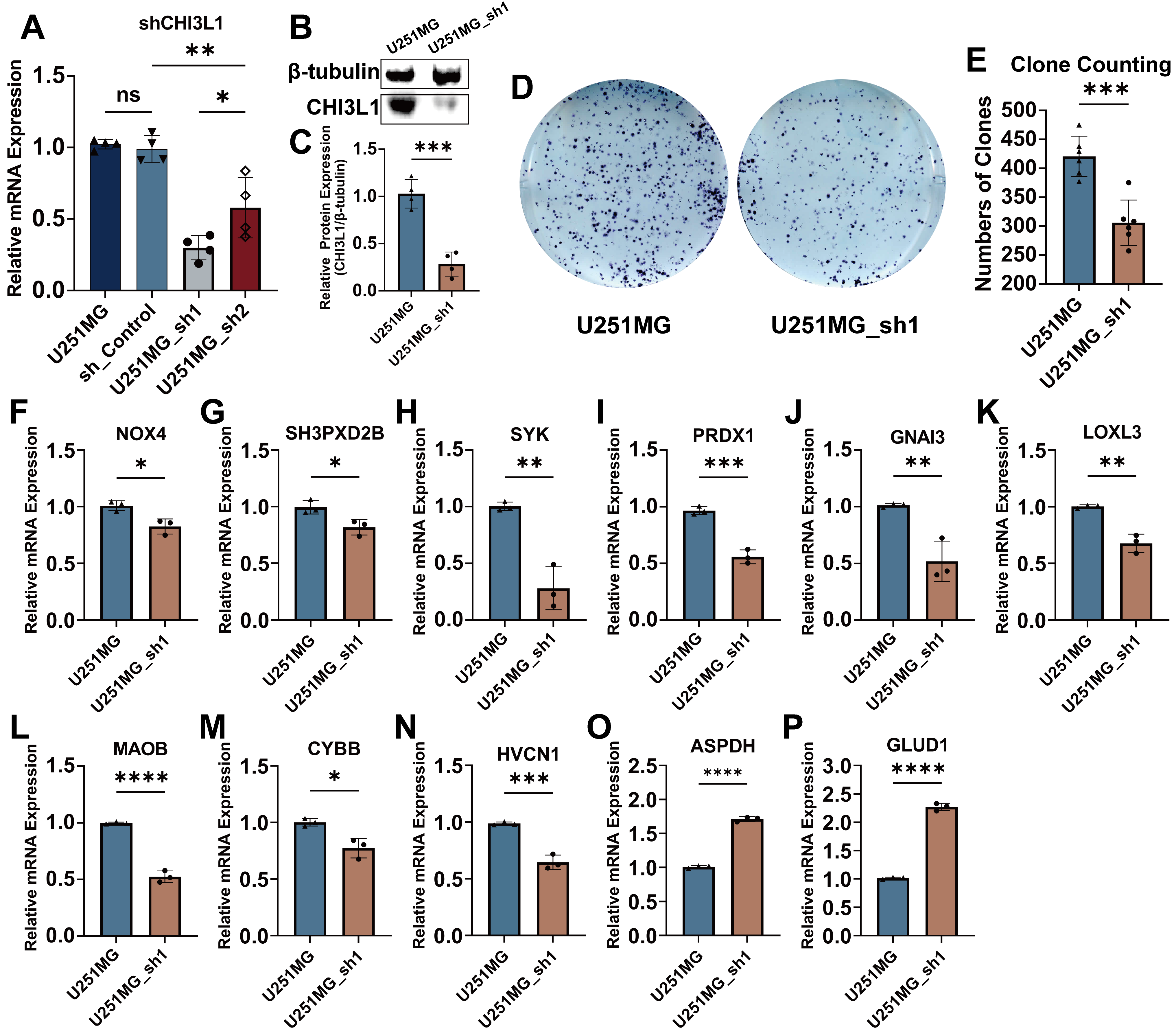

Supplement: Supplementary file 1 [file ijms-25-07094-s001.zip › Supplementary Materials/Supplementary Figure S1/Supplementary Figure S1.png]
